# Environmental approaches to controlling *Clostridioides difficile* infection in healthcare settings

**DOI:** 10.1186/s13756-023-01295-z

**Published:** 2023-09-07

**Authors:** Philip C. Carling, Michael F. Parry, Russell Olmstead

**Affiliations:** 1 Carney Hospital, Boston, MA USA; 2 Stamford Medical Center, Stamford, CT USA; 3 Trinity Health, Lavonia, MI USA

**Keywords:** *Clostridioides difficile*, Hospital onset *Clostridioides difficile* infection prevention, Disinfection cleaning, Optimized cleaning performance, Sporicidal disinfectant, Healthcare-associated infections

## Abstract

As today’s most prevalent and costly healthcare-associated infection, hospital-onset *Clostridioides difficile* infection (HO-CDI) represents a major threat to patient safety world-wide. This review will discuss how new insights into the epidemiology of CDI have quantified the prevalence of *C. difficile* (CD) spore contamination of the patient-zone as well as the role of asymptomatically colonized patients who unavoidable contaminate their near and distant environments with resilient spores. Clarification of the epidemiology of CD in parallel with the development of a new generation of sporicidal agents which can be used on a daily basis without damaging surfaces, equipment, or the environment, led to the research discussed in this review. These advances underscore the potential for significantly mitigating HO-CDI when combined with ongoing programs for optimizing the thoroughness of cleaning as well as disinfection. The consequence of this paradigm-shift in environmental hygiene practice, particularly when combined with advances in hand hygiene practice, has the potential for significantly improving patient safety in hospitals globally by mitigating the acquisition of CD spores and, quite plausibly, other environmentally transmitted healthcare-associated pathogens.

## Introduction

**Fig. 1:**
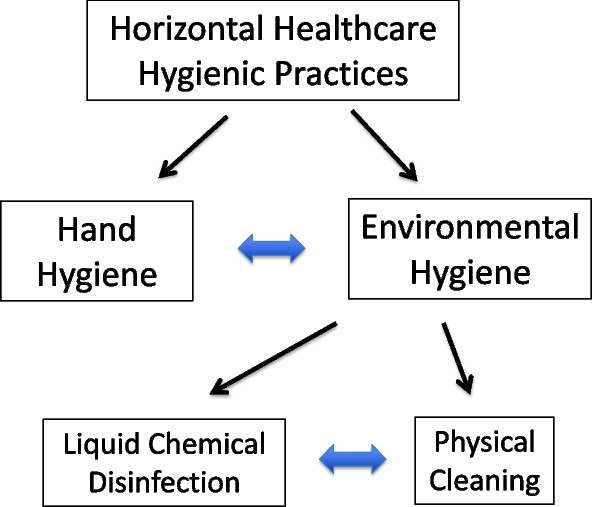
The elements of Horizontal Healthcare Hygienic Practices. The blue arrows represent the interdependence between the elements

As noted by Peters in 2022, healthcare-associated infections (HAI) are one of the greatest threats to patient safety worldwide [1]. As a result of epidemiologic and microbiologic studies over the past decade, it has become increasingly evident that interventions to mitigate environmental surface pathogen contamination constitute an important component of (HAI) prevention. Unfortunately, precisely defining how the impact of various surface cleaning interventions and concomitant hand hygiene practice can be quantified to develop clinically sound implementation science has yet to achieved [2, 3]. Despite such ongoing challenges it is important to recognize that environmental hygiene represents a critical element of what Wenzel and Edmonds defined as “horizontal interventions” that are central to mitigating a wide range of HAIs (Fig. 1) [4, 5]. These approaches aim to reduce the risk of infections caused by a broad range of pathogens by the implementation of standard practices that are effective regardless of patient specific conditions [6]. In contrast to the horizontal interventions, “vertical interventions” are pathogen and/or condition specific. While vertical and horizontal approaches are often complementary, there is evolving evidence that horizontal interventions in endemic situations may represent a best use of HAI prevention resources [6, 7]. As noted in Fig. 1, Healthcare Hygienic Practice consists of interventions which have traditionally been addressed separately, but as will be discussed below, their effectiveness in clinical settings is highly interrelated and interdependent.

The burden of healthcare associated *Clostridiodes difficile* Infection (CDI), coupled with the expectation that improved environmental cleaning could prevent these infections, has led to extensive efforts to mitigate transmission risk within healthcare settings since 1981 when Fekety et al. [8] documented widespread healthcare environmental contamination of surfaces, both near and more distant from patients with CDI. Although numerous quasi-experimental studies substituting dilute bleach for non-sporicidal disinfectants have reported a reduction in healthcare-associated CDI (HO-CDI) during outbreaks, efforts to effectively mitigate environmental transmission of *Clostridiodes difficile* (CD) spores in endemic settings has been ineffective [9–12]. New insights into the healthcare epidemiology of HO-CDI and new approaches to mitigating environmental transmission will be discussed in detail in this review.

## Global and healthcare epidemiology of *Clostridioides difficile* infections

### Global epidemiology

Although accurate assessments of global trends in CDI prevalence are challenged by variations in diagnostic methods as well as resource limitations impeding surveillance activities [2, 13], the world wide epidemiology of CDI has been characterized by rapidly evolving shifts in prevalence of disease [14]. A recent review of regional differences in (CDI) noted that global infections have been slowly decreasing between 2 and 4% per year through 2015 in most European countries while Asia has shown increasing trends through 2014 primarily due to increases in western Asia countries including Turkey and Israel [15]. In England declining rates of infection with ribotype 027 fell by half between 2007 and 2010, likely due to a concurrent reduction in fluroquinolone use [2]. While the overall trend toward decreasing CDI in Europe is of note, between 23 and 66% of cases in a range of European countries [2] and 50–60% of cases in Australia were found to be under diagnosed due to a lack of clinical suspicion and suboptimal laboratory methods [16].

In the United States, CDI rates had been showing a gradual decrease during the decade prior to the COVID pandemic, primarily due to decreases in HO-CDI [17]. Several factors would appear to have contributed to the declining incidence including antimicrobial stewardship [18], better diagnostic stewardship [19] and reimbursement negative incentive programs [20]. While some facilities experienced increases in HO-CDI early in the COVID pandemic, recent more extensive studies have failed to document a significant trend in CDI rates [21, 22].

### Evolving insights into healthcare environmental *Clostridioides difficile* epidemiology

Given the extremely low inoculum necessary to cause infection [23] and the fact that CD spores on environmental surfaces have a basically indefinite ability to remain viable decreasing only 0.5 log in 14 months [24] it is not surprising that surfaces contaminated with CD spores have a role in CD transmission. Recent studies have clarified and quantified many aspects of the environmental epidemiology of CD in hospitals (Table 1).

**Table 1:** The elements of *Clostridioides difficile* healthcare epidemiology

Elements of Clostridioides difficile Environmental Epidemiology	
1. At the time of hospitalization 10.6% of patients (range 2.8–21%) are CD carriers	Ref: [25–35]
2. During hospitalization 12.5% of patients (range 2.9–21%) are CD carriers	Ref: [25, 36–41]
3. Transmission of CD spores to environmental surfaces is associated with:	Ref: [46–48]
Patients with acute infection	
Patients recovering from acute infection	
Asymptomatic CD colonized patients	
4. Treatment does not decrease ongoing environmental spore contamination for more than a month	Ref: [49]
5. Wide spread surface contamination far from known CD infected patients	Ref: [35, 36]
6. Increased Cleaning and disinfection result in:	Ref: [99]
Decreased surface and hand contamination	
Decreased CD acquisition	
7. Genomic confirmation of the role of asymptomatic CD carriers in transmission	Ref: [37, 42–45]
8. Acquisition of CD from a prior room occupant is significantly dependent on the prior room occupant receiving antibiotics	Ref: [52, 53]

As noted in Elements 1 and 2, recent studies have shown that a substantial proportion of all acute care patients are colonized with CD either at the time of admission (average incidence density 10.6%, range 2.8–21% [25–35] or during their hospitalization (average prevalence density 12.5%, range 2.9–21%) [25, 36–41]. As a result, approximately 11% of hospitalized acute care patients present an ongoing risk of CD transmission to the environment and susceptible patients. Genomic epidemiology has now confirmed the environmental transmission of spores from these patients to other patients [37, 42–45]. As noted in Element 3, patients recovering from acute CD infection are associated with significant transmission of spores to their environment [46–48]. This issue was carefully analyzed in a multi-site study by Davies et al. in 2020 which evaluated the impact of treatment for CD infection on patient-zone environmental contamination [49]. Treatment of CD infection with metronidazole, vancomycin or fidaxomicin similarly decreased the proportion of patients with positive stool cultures from 100 to 35% immediately after treatment. Following treatment, the rate rebounded to 80–90% by 2–4 weeks later. And although there was a decrease in the proportion of environmental sites contaminated with CD spores from 36% before treatment to 20% immediately following treatment, environmental contamination by these patients was still at 27% four weeks after completing treatment, confirming the significant ongoing risk of transmission of CD to other patients and healthcare workers by patients who had completed treatment for CDI. These studies confirm substantial levels of environmental contamination, but they may actually under-estimate the problem. A recent study using PCR technology confirmed a tenfold increase in the frequency of surface contamination in comparison to direct culture [50]. In 2015 Kundrapu, documented that spore shedding and near patient environmental contamination with CD spores was substantially increased when asymptomatic patients colonized with CD were administered antibiotics [51]. The clinical relevance of this phenomenon was subsequently clarified by Freedburg et al. [52].They analyzed a cohort of more than 100,000 patients who sequentially occupied a given hospital bed and found that independent of the prior room occupant’s CDI status, administration of antibiotics to the prior bed occupant was the most significant factor associated with an increased risk of the next bed occupant developing CDI. The same phenomenon was also identified by Dowling Root in 2021 [53]. In this study of 17,285 patient room occupancies the risk of HO-CDI was significantly associated with prior room occupant antibiotic usage (Odds Ratio 2.37, *p* < .001). The results of these two large studies, can only be explained by recipient acquisition of residual CD spores asymptomatically shed onto patient-zone surfaces by the preceding room occupant.

## Mitigating *Clostridiodes difficile* spore transfer from environmental surfaces

### Chemical disinfection

Chlorine-based disinfectants, particularly diluted commercial grade bleach has been used extensively for terminal cleaning of CDI patient rooms [54]. Unfortunately, physical damage associated with the use of these disinfectants precludes their daily use for all high-touch patient-zone surfaces. Fortunately, we now have broad-spectrum sporicidal agents that are at least as effective as bleach, are not associated with significant damage to surfaces, and are not associated with potentially toxic residuals during either their use or disposal [55, 56]. These hydrogen peroxide/peroxyacetic acid formulation chemistries are rapidly sporicidal and are also effective against *Candida auris,* healthcare-associated pathogens (HAPs)*,* norovirus and other viral pathogens, including corona viruses [57]. While these chemistries have been widely used and their effectiveness well validated, other non-chlorine based sporicidal agents are becoming available.

### Surface disinfection technologies

Despite in vitro studies confirming the resistance of CD spores to UV light, programs incorporating UV technology have been reported to have impacted HO-CDI rates in hyper-endemic settings [58]. In contrast, several more recent reports of such programs failed to show an impact on endemic HO-CDI rates [12, 59–62] and a multi-year cluster randomized crossover control trial found that the daily UV supplemented intervention did not reduce either HO-CDI rates or VRE transmission [63]. These results have now been further supported by a cluster-randomized sham-controlled double blinded crossover trial of a UV program, by Kaye et al. involving 25,732 patient room cleanings. It failed to show an impact on HO-CDI rates, which were actually higher in the sham UV treatment arm (p 0.53) [64]. Furthermore, the prevalence of *E. coli* and *Staph. aureus* contamination of high-touch patient-zone surfaces was unchanged [65, 66]. Uncontrolled studies utilizing hydrogen peroxide vapor technology as part of terminal cleaning of CDI patient rooms have appeared to be associated with a decrease in HO-CDI [67–69] but they were lacking confounder assessment. In addition, logistical challenges in delivering the treatment may hinder the use of this technology beyond CDI isolation room terminal cleaning [58].

## A programmatic approach to optimizing environmental hygiene to mitigate HO-CDI

### Evaluating disinfection cleaning

The importance of physically removing visible dirt and soil from surfaces in hospitals has been recognized for more than 150 years [70]. Consequently, all acute care hospitals have policies and procedures to define the role of environmental services personnel for cleaning patient-zone surfaces. Environmental services (EVS) managers and infection preventionists had implemented joint visual inspection of surfaces in patient care areas well before the CDC recommended that hospitals clean and disinfect “high-touch surfaces” in 2003 [71]. The CDC further recommended that hospitals “monitor, (i.e., supervise and inspect cleaning performance) to assure consistent cleaning and disinfection of surfaces in close proximity to the patient and likely to be touched by the patient and healthcare professionals” in 2006 [72]. Unfortunately, the intrinsically subjective nature of such monitoring along with its episodic and deficiency-oriented features limit its ability to accurately assess the thoroughness of day-to-day cleaning activity. Preliminary studies documenting patient zone surface contamination with HAPs raised concerns that cleaning practice should be improved [73]. It was not until actual cleaning practice was objectively monitored, initially using a covert visual monitoring program [74] and later with covertly applied fluorescent markers, that actual cleaning practice was objectively evaluated [75, 76]. Evaluations were done in a standardized manner with a metered fluorescent marking system (DAZO™ Ecolab, Inc., St. Paul, MN). The outcome measured was the actual thoroughness of cleaning expressed as the “thoroughness of disinfection cleaning” or”TDC” [77]. Given the accuracy of the metered fluorescent markers to objectively and reproducibly identify opportunities to improve cleaning thoroughness, process improvement interventions based on structured educational activities and direct performance feedback to EVS staff were shown to be highly effective in improving cleaning outcomes [78, 79]. Despite the challenges the EVS staff contend with [80], published reports of these programs have now confirmed the effectiveness of such programs with the TDC improving from 40–60% to 80–90% or higher for at least 3 years [79, 81]. Most recently Parry (2022) evaluated the sustained impact of a structured ongoing monitoring and feedback program to optimize patient-zone disinfection cleaning in a 305 bed acute care hospital over 10 years [82].

**Fig. 2:**
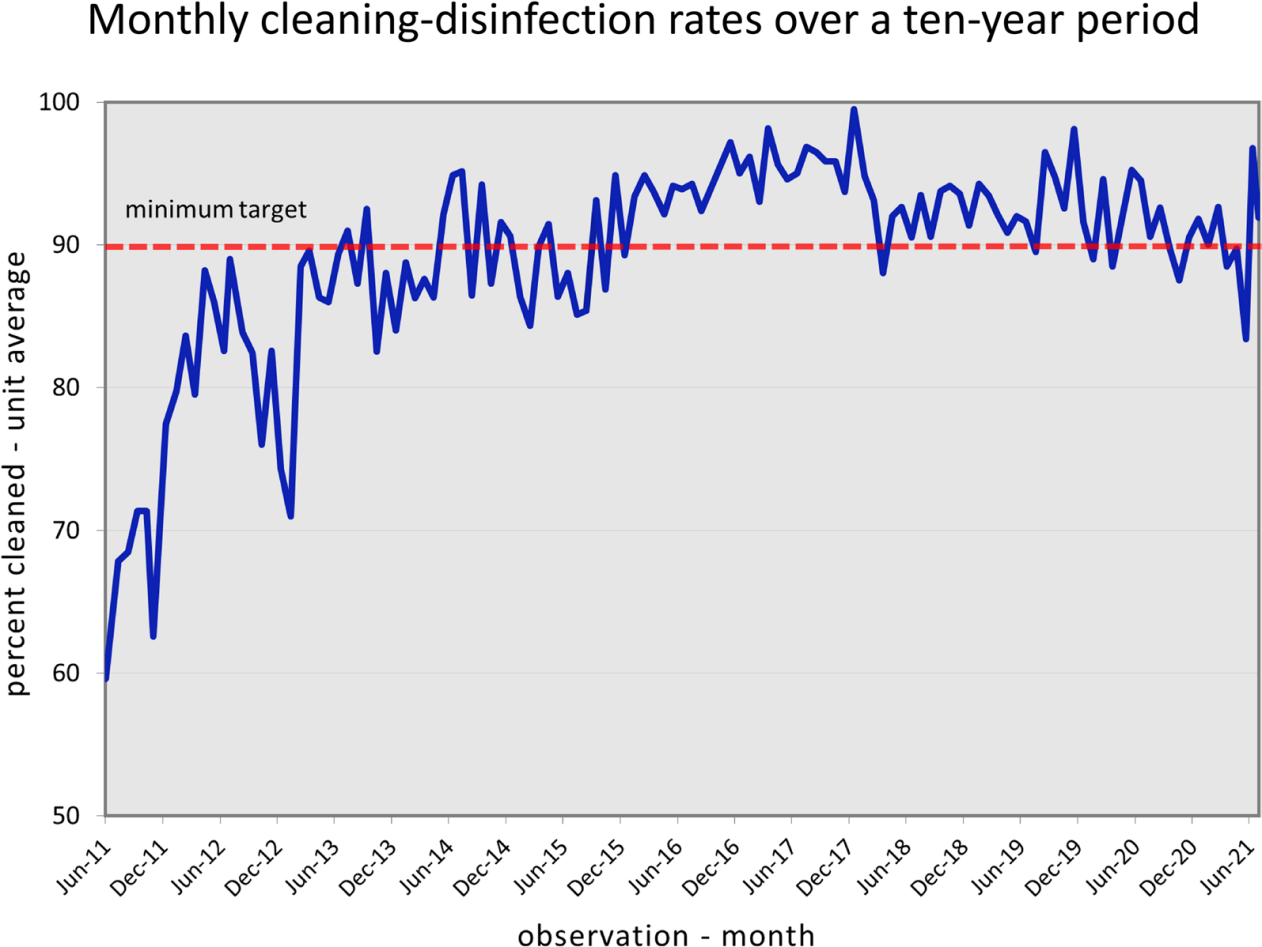
The thoroughness of disinfection cleaning as objectively documented by the standardized florescent marker monitoring program

The cleaning/disinfection performance of the EVS staff was covertly measured by specially-trained infection prevention nurse liaisons to minimize bias and telegraphing surface marking sites. As noted in Fig. 2, cleaning performance improved from a baseline TDC of 60% to greater than 80% over the first year of the program. Subsequently most quarterly rates were at or above the 90% minimum target during the final six years reported. The process improvement success of programs related to patient zone disinfection cleaning had also been realized with respect to the operating theatre setting [82, 83].

“Tools For Evaluating Environmental Cleaning: The Guidance Environmental Cleaning Procedures” As a result of published evidence supporting objective monitoring to evaluate surface cleaning processes, the CDC developed the guidance “Options for Evaluating Environmental Cleaning” in 2010 and updated it in the Guidance “Best Practices for Environmental Cleaning Procedures” in 2020 [77, 84]. which recommends the use of a fluorescent marker-based performance monitoring program along with direct observation of cleaning practice.

Studies in the United States and abroad during the past 20 years have used a specially developed fluorescent gel or “test soil” to covertly evaluate environmental cleaning in a wide range of healthcare settings [75, 76, 85–89]. These studies have utilized a standardized metered transparent gel specifically formulated for the covert evaluation of healthcare surface cleaning. While non-standardized fluorescent powders and lotions have been used in a non-covert manner for education [90], other studies [89, 91] demonstrated that these substances visibility in ambient light limited their effective use in programs to objectively monitor cleaning practice as a result of their ability to induce a Hawthorne effect. In 2019 a study from Johns Hopkins compared the clinical use of the metered applicator with a standardized fluorescent gel to a cotton swab applicator with a non-standardized fluorescent gel and found that the metered applicator provided a more accurate assessment of cleaning practice. The authors concluded that, “Infection control programs implementing evaluation of environmental cleaning programs should carefully consider the type and method of applying fluorescent gel marks to standardize and optimize the measurement of fluorescent gel removal” [92, p.796].

ATP bioluminescence technology detects the presence of organic material, including viable and non-viable bioburden, on surfaces. Although their ease of use led to their use to attempt to quantify healthcare surface bioburden, the high sensitivity of the system to non-microbiologic and non-viable organic matter and its relative insensitivity to some healthcare-associated pathogens has now been clarified [93, 94]. As noted by Mulvey, et al. in a detailed evaluation of the ATP technology, “Sensitivity and specificity of 57% (with the ATP tool) means that the margin for error is too high to justify stringent monitoring of the hospital environment (with ATP technology) at present” [95, p.29]. As noted in the CDCs Guidance Best Practices for Environmental Cleaning in Healthcare facilities (2020): (Section 4. Tables 29 and 30) ATP technology is not recommended for evaluating cleaning performance [84].

An important requirement for monitoring and process improvement programs relates to the need for them to have a successful validation component. As noted in the 2010 CDC guidance, “It is important that the monitoring be performed by hospital epidemiologists, infection preventionists or their designees who are not part of the actual EVS cleaning programs. Such an approach, as discussed previously, assures the validity of the information collected” [77 (Appendix B, p.1, 82]. The importance of this issue was confirmed in a study which found that when EVS managers monitored the discharge room cleaning, they documented an average TDC score of 82.5% while a research team covertly evaluating the same two hospitals documented an average score of 52.4% [96]. Given the fact that neither the Joint Commission or the World Health Organization consider self-monitoring of hand hygiene practice to be acceptable, it seems reasonable that a similar expectation should be applied to monitoring disinfection cleaning activities.

## Implementing the 2020 CDC guidance: core components of environmental cleaning and disinfection in hospitals

In October 2020 the CDC published a guidance document to provide hospitals with a detailed roadmap for the development of programs to optimize all aspects of patient-zone environmental hygiene because “maintaining a clean hospital environment and minimizing the presence of hospital pathogens is critical for keeping patients safe” [97, p.e1].

**Fig. 3:**
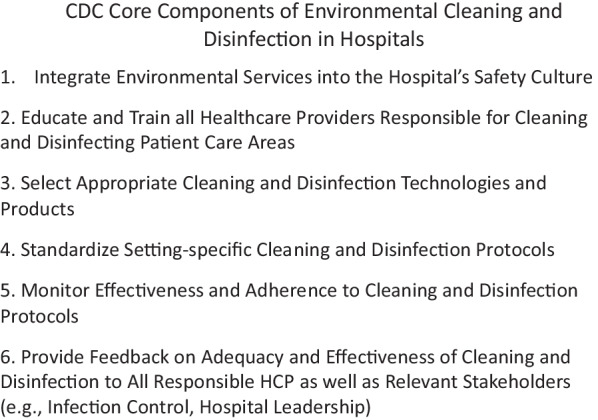
The CDC core elements of environmental cleaning and disinfection in hospitals

The six individual “core components” (Fig. 3) and the specific recommendations within each of the strategies detailed in the document specify what “every healthcare facility should consider to ensure appropriate environmental cleaning and disinfection” [98, p.e1]. While not specifically discussed in the document, describing the (EVS) staff involved in patient-zone cleaning and disinfection as “healthcare personnel” represents an acknowledgment of the relevance these activities have to safe patient care. Taken together, these Core Components provide a detailed, clearly structured, comprehensive template, based on implementation science studies over the past 20 years, to optimize all aspects of environmental hygiene practice for acute care hospitals which can also be adapted to a wide range of patient care settings [81].

## Assessment of the potential impact of thorough daily sporicidal disinfection cleaning in mitigating HO-CDI

Given the fact that general use of sporicidal disinfectants on patient-zone surfaces is now feasible, it is possible to quantitively assess the impact of daily sporicidal disinfection cleaning of all high-touch patient-zone surfaces in mitigating CD transmission. This approach was initially evaluated in a single-site, quasi-experimental study in 2016 [98].

**Fig. 4:**
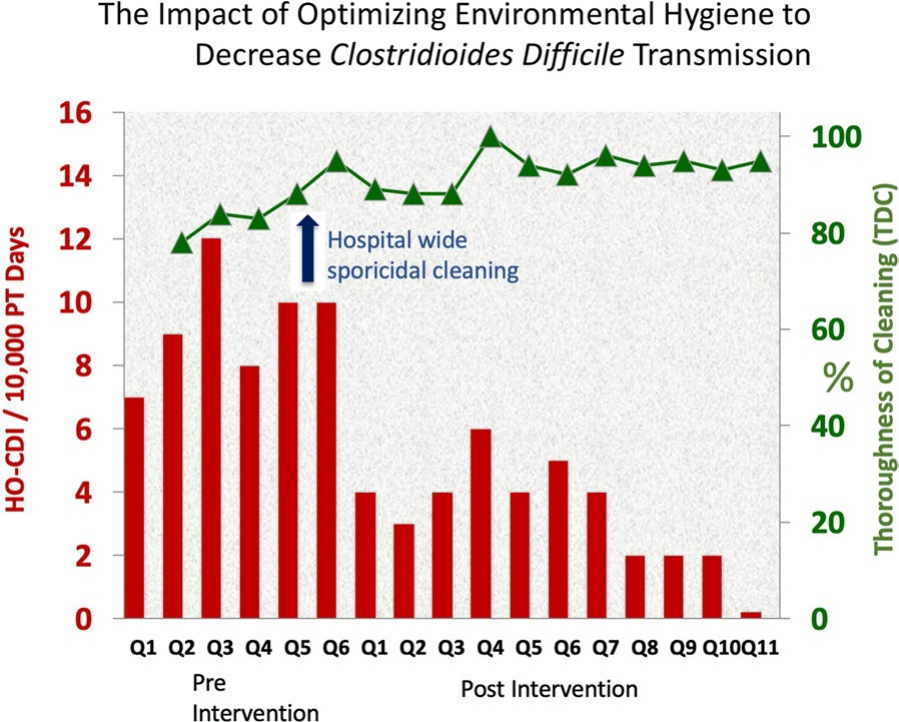
The impact of optimizing environmental hygiene to decrease *Clostridioides difficile* transmission in a single hospital over two and one half years

As noted in Fig. 4, during the 33-month intervention period, thoroughness of disinfection cleaning (TDC) rapidly improved from 81 to 92% and remained greater than 88% during the remainder of the study (*P* = .01). HO-CDI rates fell significantly during the intervention period from an average of 8.9–3.2/10,000 patient-days (*p* = 0.0001, 95% CI 3.48–7.81).

The clinical impact of implementing daily, hospital-wide sporicidal disinfectant cleaning of all patient-zone surfaces was further evaluated using a control group validated, quasi-experimental, interrupted-time series analysis employing a group of eight acute care hospitals [99]. These hospitals had stable endemic Standardized Infection Rates (SIRs) (Mean 1.03 for the group) during an 18-month pre-intervention period. The intervention hospitals within the healthcare system studied ranged in size from a 532-bed tertiary care hospital to a 44-bed regional critical access hospital (mean 257 beds). Nine randomly selected hospitals from the same system that had not enrolled in a standardized (EVS) process improvement program served as controls. (mean 266 beds). Thoroughness of cleaning was programmatically monitored in accordance with the 2010 CDC guidance[77] using a standardized metered fluorescent marking system (DAZO™ Ecolab, Inc., St Paul, MN).

**Fig. 5:**
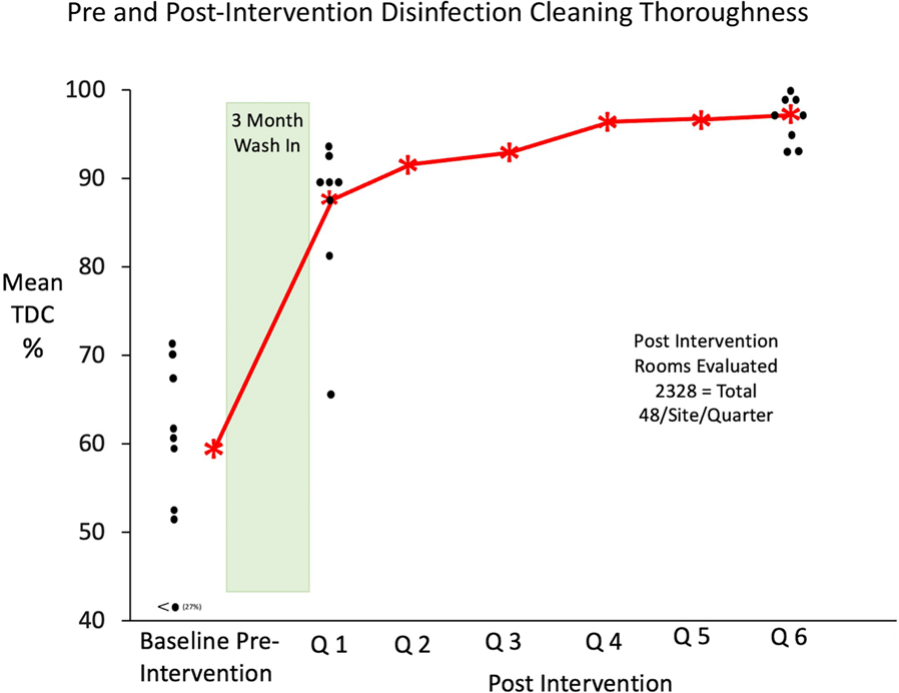
Toroughness of Cleaning in 8 Intervention Hospitals

As noted in Fig. 5, TDC following educational activities during the 3-month wash-in period improved rapidly from 59 to 88%. With the use of ongoing quarterly performance feedback, cleaning thoroughness continued to improve over the next 5 quarters and at 18 months the TDC was 93.6% for the group (Range 91–96%, 95% CI 45–24%, *p* < 0.0001).

**Fig. 6:**
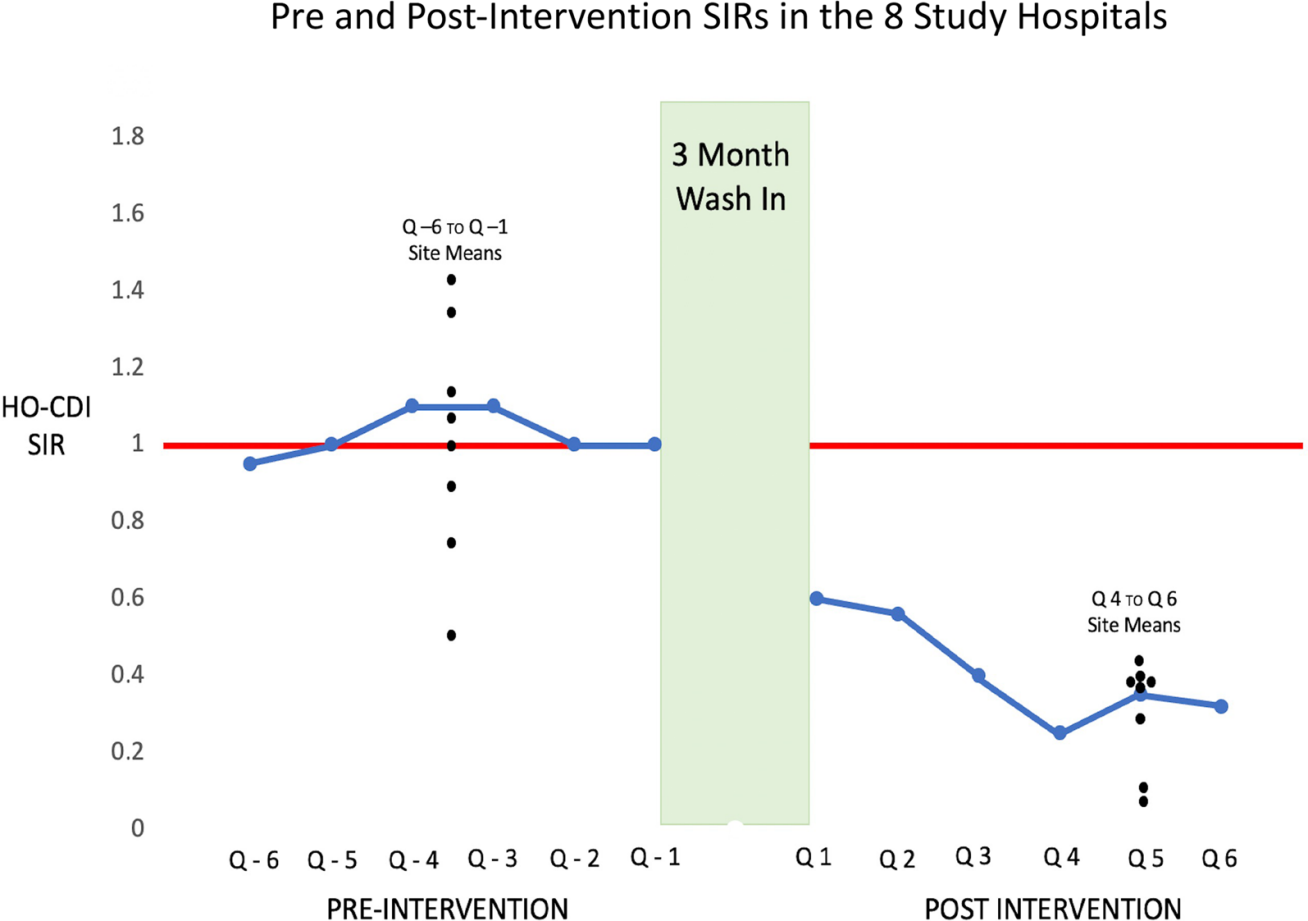
The trend in HO-CDI SIR pre and post-intervention

As noted in Fig. 6, mean group HO-CDI SIRs ranged from 0.49 below to 1.42 above a mean of 1.03 during the 18 months prior to project implementation. In quarter-1 following wash-in, all sites documented a decrease in HO-CDI to a mean SIR of 0.6 (95% CI 0.13–0.75, *p* = 0.009). Over the next 5 quarters, the HO-CDI SIR continued to decrease stabilizing during the last three quarters evaluated to a mean SIR of 0.4 (95% CI 0.13–0.75, *p* = 0.009).

**Fig. 7:**
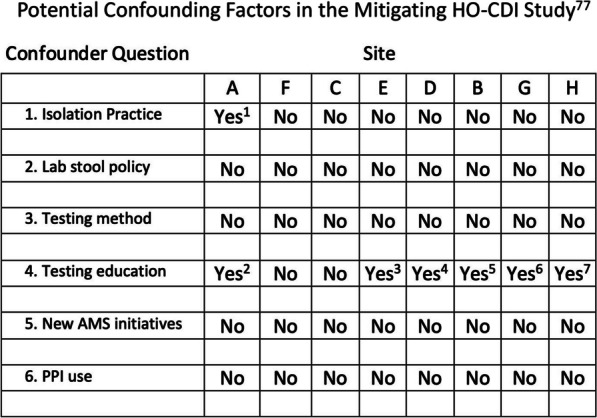
Evaluation of potential confounding influences. 1. Q3 pre-intervention year “Enhanced contact precautions for CD positive patients was implemented”. During the first 6 to 9 months of the pre-intervention period these sites implemented nursing education to clarify the importance of early stool specimen collection in patients with diarrhea

As outlined in Fig. 7 seven potentially significant confounders were evaluated pre-and post-intervention and were found not to have had an impact on the results. Using the control hospitals in an adjusted difference-in-differences analysis, the intervention was associated with a 0.55 reduction (95% CI − 0.77 to − 0.32) in HO-CDI (*p* < 0.001; or a 50% relative decrease from a baseline SIR of 1.03). The study represents the first multi-site, quasi-experimental study with control hospitals to evaluate a daily, hospital wide, performance optimized, sporicidal, disinfection cleaning on HO-CDI. While a randomized controlled trial could further clarify and quantify the results of this intervention, such an undertaking would require considerable resources as well as the need for sites to defer implementing potentially effective design elements of the intervention.

**Fig. 8:**
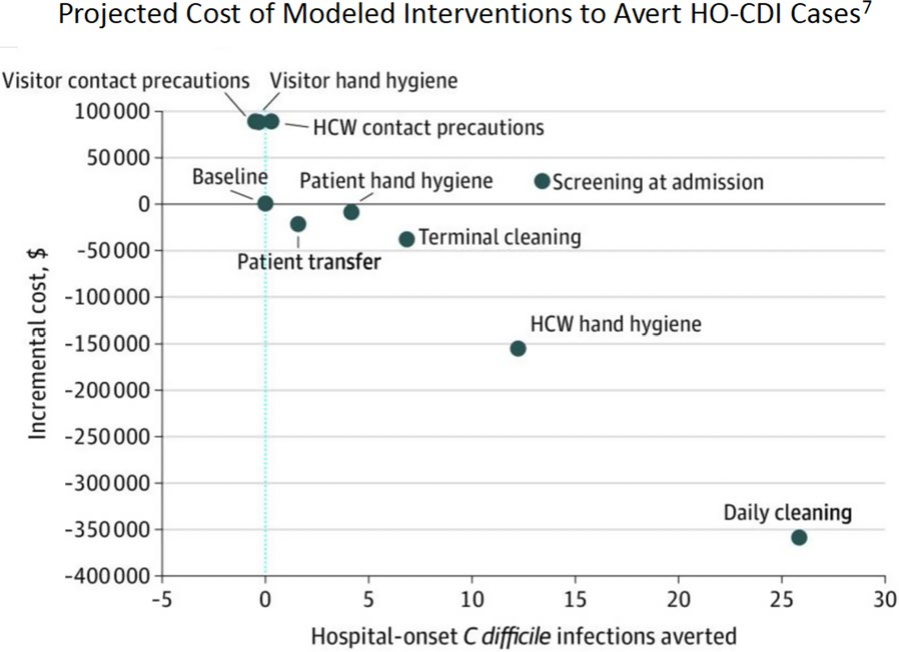
Evaluation of the modeled cost (cost-avoidance) associated with interventions to mitigate HO-CDI

Given the challenges of a randomized trial, it should be noted that the open-access published, agent-based modeling study by Barker, et al. evaluating the impact of multiple single and bundled interventions on HO-CDI prevention found that the single most clinically effective and cost-effective intervention was daily sporicidal cleaning of all patient zone surfaces as depicted in Fig. 8 [7]

Furthermore, quantitative input analysis of the model found only a limited additional incremental benefit from increasing modeling parameters of thoroughness of cleaning from an “enhanced level” (80% TDC) to an “ideal level” (94% TDC), suggesting that daily patient zone sporicidal cleaning could have a substantial impact on CD transmission when TDC is lower than those achieved by the intervention group of hospitals discussed above [7]. Most recently, this agent-based simulation model was used by Scaria, to compare it with primary observed data from a 426 mid-western, US hospital over 6 years in order to compare the predicted HO-CDI rate to the observed rate between 2013 and 2018.

**Fig. 9:**
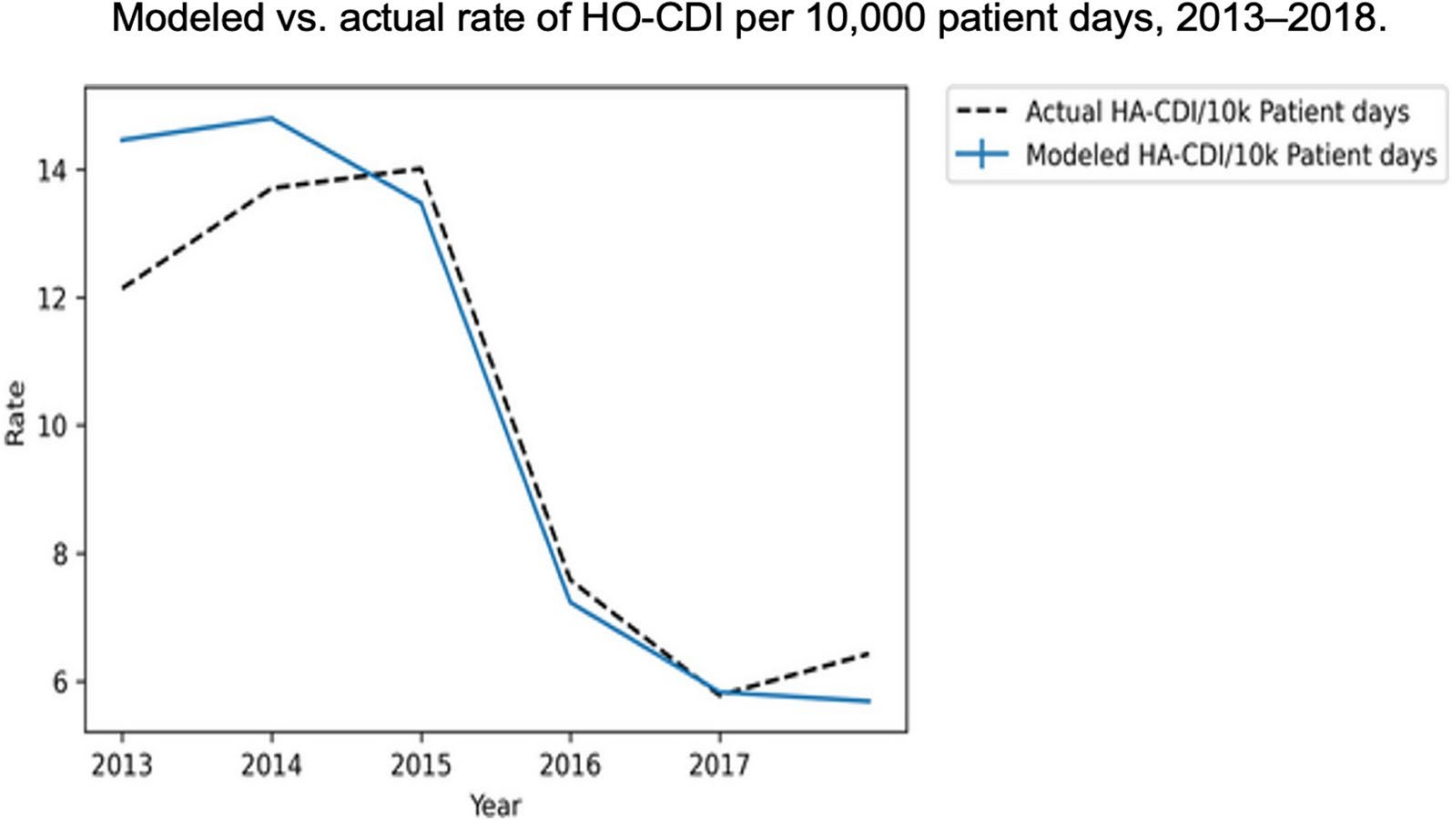
Comparison of the modeled and observed HO-CDI over 6 years

As noted in Fig. 9.,the trends in both the modeled and actual rates were nearly identical following implementing “increased infection control measures” namely, daily patient-zone sporicidal disinfection cleaning and improvement in the TDC from 56% in 2013 to 79% in 2017 and 2018 [100]. Of note, the decrease of 46% in HO-CDI, both predicted and observed, was similar to the decrease of 50% documented in the eight hospital study previously discussed.

## Additional benefits of mitigating CD environmental transmission

### Collateral microbiological benefits

Over the past several years there has been increasing documentation of the potential and actual role of surfaces in the near patient environment being relevant in HAI epidemiology.

**Fig. 10:**
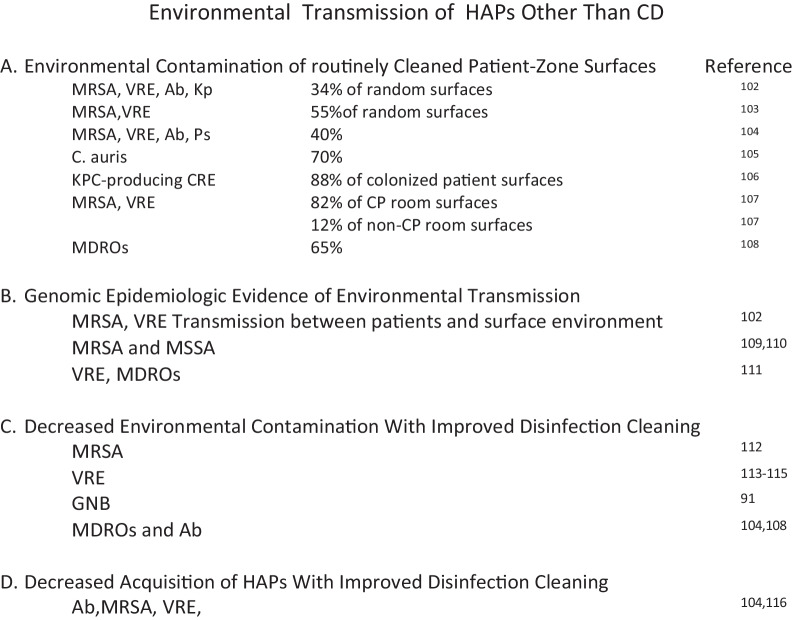
Studies which have clarified the potential for optimized patient-zone disinfection cleaning to mitigate the transmission of healthcare -associated organisms from environmental surfaces

As noted in Fig. 10, [101–116] patient-zone environmental surfaces are frequently contaminated with a wide range of HAPs. While the frequency of contamination is greatest close to patients, genomic epidemiology has confirmed more distance spread [11, 116]. While documenting high level CRE contamination (88% of surfaces) associated with colonized patients, the study by Shams also found that 80% of all contamination was associated with 20% of colonized patients which they characterized as “super shedders” [101, 117] Although many of the HAI-associated pathogens in Fig. 10 are effectively killed by quaternary-ammonium compounds, or accelerated hydrogen peroxide the use of hydrogen peroxide-peroxyacetic acid chemistries for CD mitigation would allow for highly effective disinfection of surfaces harboring Candida auris, norovirus and quaternary-ammonium resistant *A. baumannii* [118].

Finally, it should be noted that recent reports documenting widespread intra-system and inter-system transmission of carbapenem-resistant *Klebsiella pneumoniae* and the possibility that terminal patient room cleaning enhanced by a UV treatment protocol can impact the occurrence of hospital-onset bacteremia with some strains of gram negative rods suggest that there is yet much to be clarified regarding the role of patient zone surfaces in the epidemiology of many HAPs [119, 120]

### Quality of life benefits

While the acute morbidity and mortality (approximately 5%) of CDI have represented significant issues for years, analysis of more complex effects of CDI are now being quantified. As part of the agent-based modeling study previously described, Barker used a standardized quality-of-life years (QALYs) analysis and found that the impact of the daily, performance optimized (80% TDC) sporicidal disinfection patient-zone cleaning intervention in the modeled 200 bed hospital with a 1.0 SIR was associated with a savings of 36.8 QALY s annually for such a program [7].

In addition to QALYs lost as a result of CDI, the illness has a substantial adverse impact on patient reported quality of life. This phenomenon was recently quantified in a controlled study by Han (2022) using a health-related quality of life 32 element questionnaire [121]. The study found that patients hospitalized with CDI developed a quantifiable negative impact on multiple physical and mental health measures. Of note was a particularly adverse impact of recurrent CDI (10% of patients) on the quality-of-life parameters measured.

### Economic benefits

While the direct impact of CDI in terms of morbidity and mortality has been well documented, several in-depth population based studies published between 2011 and 2022 have analyzed the economic costs of CDI [122–125]. In considering the impact of these costs, it is critically important to note that a substantial proportion of the total costs per case are not reimbursed by commercial insurance, Medicare or Medicaid in U.S. hospitals. As summarized in Fig. 10, four studies have specifically evaluated the cost of HO-CDI (Table 2).

**Table 2:** Population based studies which have evaluated the average attributable and non-reimbursed cost of HO-CDI in US hospitals

Population based studies on the cost of HO-CDI				
Author	Study period	Publication date date	Total attributable cost per case (USD)	Non-reimbursed Cost Per case (USD)
Shorr [122]	2008–2010	2022	$28,050	Not evaluated
Mollard [123]	2012–2016	2019	$27,122	Not evaluated
Sahrmann [124]	2011–2017	2022		$14,257
Yu [125]	2012–2019	2022	$28,762	$13,476

These matched controlled studies evaluated between 6000 and 60,000 HO-CDI cases over 2–7 years. The three studies which evaluated total cost found almost identical results while the two studies which evaluated non-reimbursed costs also found highly similar costs of $14,257 and $13,476 or approximately 50% of the total cost. This proportion of non-reimbursed costs is also consistent with an earlier study of 272, 143 hospitalizations which found that 65% of the cost of HO-CDI was not reimbursed by Medicare payments [126]. Although not stratified to identify costs for HO-CDI, Magee in 2015 determined the excess cost for hospitalized CDI patients to be $24,408 based on data from 2009 to 2011 [127]. In a similar study Zhang in 2019 found excess total cost per case of $24,205 [128].

As modeled by Barker [7] using data from published studies, a 200 bed hospital with a HO-CDI rate at the national average and a non-reimbursed cost per case of $12,313 was projected to have an annualized savings of $358,268 as a direct result of implementing daily hospital-wide patient zone sporicidal disinfection cleaning at a 70% TDC. Based on this modeling and the population based studies discussed it is likely that the 8 intervention hospitals previously discussed (average size 258 beds, HO-CDI rate at the national average pre-intervention) realized an annualized savings of approximately $3.7 million per year during the last 12 months of the study.

Based on this research, it is feasible for a hospital to project the yearly non-reimbursed cost of HO-CDI. Taken together, these studies support the likely probability that each case of HO-CDI had a non-reimbursable cost of approximately $12,000 between 2008 and 2019. While the current cost can be estimated based on these studies Yu and co-authors in 2019 noted that, “As CDI management evolves, the already substantial per-patient health care costs and health care utilization associated with CDI are likely to increase” [125, p.1].

## Environmental hygiene and hand hygiene: an integrated approach

Over the past several years it has become increasingly evident that infection prevention initiatives focused on optimizing hand hygiene have not realized their hoped-for impact on healthcare-associated pathogen (HAP) transmission in well-resourced healthcare settings [129–133]. Accepting our inability to quantify the absolute risk of pathogen acquisition directly from healthcare workers’ hands, there is good circumstantial evidence that such transmission accounts for a substantial proportion of HAP transmission. Indeed, it has become widely accepted that hand hygiene, as noted by Palamore, is “critically important for the prevention of HAIs” [134] (p.8).

Given the fact that patient zone surfaces not contaminated by HAPs cannot be a source of pathogen transmission even in the absence of hand hygiene, further consideration must be given to viewing both environmental hygiene and hand hygiene as being interdependent interventions since these two interventions are intrinsically interdependent, they represent what can be termed “hygienic practices” (Fig. 1.).

## Conclusions

In discussing the 2022 Clean Hospitals Healthcare Cleaning Forum, Peters noted that “Healthcare environmental hygiene has become recognized as being increasingly important for patient safety and the prevention of HAIs” [135, p.1].

Given the fact that HO-CDI is the most frequent HAI today, representing 56% of NHSN-reported HAIs in US hospitals (ref) and likely so globally, its mitigation is clearly critical [136]. In light of our recent greatly clarified understanding of the healthcare epidemiology of HO-CDI; implementation of new antibiotic and testing stewardship programs; the development of new potent sporicides which can be used on a daily basis for patient-zone disinfection cleaning; and the extensive documentation that such cleaning can be sustainably optimized with ongoing education and objective, quantitative performance monitoring and feed-back, there is reason to believe that great reductions in HO-CDI are feasible, particularly when hand hygiene is also optimized.

While studies incorporating genomic epidemiology will be needed to quantify the impact of HO-CDI mitigation on other HAIs, the documented mitigation of MRSA and VRE acquisition with moderately improved TDC and the environmental epidemiology of a wide range of HAPs suggests that there will be collateral benefits of mitigating HO-CDI [74, 115].

## Data Availability

The manuscript will be available as open-access and through the corresponding author.
